# Prof. Owen’s Conclusions on the Origin of Species and Nature of Life

**Published:** 1869-02

**Authors:** 


### Miscellaneous.

### Prof. Owen's Conclusions on the Origin of Species and Nature of Life.

We showed in a former article that Prof. Owen is no votary of the Divinity of Chance. On the contrary, he believes that as every individual animal passes through a succession of forms—embryonic, infantine, adult, and aged—so each group of similar animals descended from common parents, which we call "species," has an innate and fore-ordained tendency to deviate from the parental type, and to produce new forms of a more specialized character. "A purposive route of development and change, of correlation and interdependence, manifesting intelligent Will, is as determinable in the succession of races as in the development and organization of the individual. Generations do not vary accidentally in any and every direction, but in pre-ordained, definite and correlated courses."

And as with the coming in of new species, so with the extinction of old ones; if the one cannot be believed to be due to fresh acts of miraculous creation, so must the other not be considered due to occasional cataclysm or convulsion, but to the steady operation of law. One cause of extinction recognized by Prof. Owen is defeat in the struggle for existence. In 1850 he had shown this, when he said that, in a dry season, the large mammal will suffer from the drought sooner than the small one; if food be scanty, the large one will perish before the small one; if new enemies be introduced, the larger and more conspicuous will be the earlier victims; and smaller animals are, as a rule, more prolific than large ones.

As a test of the theories which account for the origin of species, Prof. Owen brings forward the coral. The species of existing *anthozoa* cannot be traced very far back; those with a flexible or with a branched calcareous axis begin only at the tertiary period, and of the genera of eocene lamellate or stony corals all the species are extinct, and have been superceded in their grand and useful operations by those now forming reefs and atolls. As we extend our researches back in time, we find generic and family types of coral polypes passing away; and that the prevalent pattern of stellate cups of rays of *six* or its multiples has superceded a similar pattern of *four* or its multiples.

Now, taking these facts, Prof. Owen asks whether a direct act of miraculous creation must be invoked to account for each successive species of coral. Such an idea he dismisses as contrary to the worthy conception of an all-seeing, all-provident Omnipotence. It is not, he says, above, but against reason.

Let us, then, assume that the modern are the direct descendants of the ancient corals, and with Prof. Owen "test the propounded explanations of their origin by secondary law." That of appetency is untenable, because a coral polype cannot exercise volition. Lamarck's creative machinery can only be applicable to creatures high enough to "want to do something." Is there any difference in the "ambient medium?" We have no knowledge that the polypes of the Devonshire or Cambrian hills worked in an ocean different from the present, or that, if different, it could change a quadripartite into a sexpartite disposition of the coral cells. The "personifying the fact of such transmutation by the term 'Natural Selection,' gives no more insight into the manner of the operations than we learn of that of the budding out of a new leg in a maimed newt by being told that it was done by the 'nisus formativus,' or by 'pangenesis.'"

Prof. Owen sums up the contrast between his own theory of "Derivation" and Darwin's theory of "Natural Selection" in few words, which we thus venture to abridge. "Derivation" holds that each species changes in time by virtue of inherent tendencies. "Natural Selection" holds that this is effected by altered external circumstances. "Derivation" sees the purpose of the Creator in the variety and beauty of creation, and the adaptation of each member of it to others, and especially man, a being capable of appreciating beauty. "Natural Selection" feels that if ornament or beauty in itself should be a purpose in creation, it would be absolutely fatal to it as an hypothesis. "Natural Selection" leaves the origin and succession of species to the fortuitous concurrence of outward conditions. "Derivation" recognizes purpose.

Prof. Owen, dismissing the old doctrines as absurd, and Darwin's pangenesis as absurder, believes to the full in what has been called "spontaneous generation," or the incessant new development of living beings out of non-living material. He sides with Pouchet and Child against Pasteur. He does not believe in "panspermism,"

or the doctrine that all the forms of life produced in decaying organic matter come from germs dispersed through the air. He prefers believing that, when the requisite material and conditions are present, other forces are resolved into vital force; and sees "the grandeur of creative power," not in the exceptional miracle of one or few original forms of life, but in the "daily and hourly calling into life many forms by conversion of chemical and physical into vital modes of force." The "CAUSE which has endowed His world with power convertible into magnetic, electric, thermotic, and other forms or modes of force, has also added the conditions of conversion into the vital mode." "Change of force forms part of the constitution of the Kosmos."

We will not follow Prof. Owen minutely in the comparison which he draws between life and magnetism, and between all the actions of living beings, from the attraction of the amœba by a bit of meat to the highest phenomena of consciousness in man; of which his conclusion is that from the magnet which chooses between steel and zinc to the philosopher who chooses between good and evil, the difference is one of degree, not of kind, and that there is no need to assume a special miracle to account for mental phenomena.

Although these ideas must fairly be called materialistic, and openly oppose the notion of an "immaterial indestructible soul," yet nothing can be further from Prof. Owen's doctrines than the *low* materialism which sees law without a law-giver, force without an author, and no God apart from matter. It must be remembered in the first place that Prof. Owen's ideas of life necessitate the belief in the perpetual presence and working of a personal God, the Lord and Giver of life; that he believes in a future life and resurrection and judgment of the dead, "on the ground of their being parts of a Divine revelation;" and that he shows (and quotes the history of the Witch of Endor and of the doubting Apostle Thomas to exemplify it) that we really are in no condition to say what is material and what immaterial. We only know of force and its effects, but (as Faraday said) as for what causes these effects we get nothing by defining them as material or immaterial. For our own parts, we must not wander into the ground of dogmatic faith, but, as regards reasonable opinion, we must say that Prof. Owen's own doctrines tell quite as much for the existence of an immortal soul as not; that the results of force must be as indestructible as matter, save by the will of God, and soul is one mode of force; and, if the matter be doubtful, we ourselves are not ashamed to be biased by the spiritual instincts of universal man, and to say, with the Pagan philosopher, "*Si in hoc erro, quod animos hominum immortales esse credam, lubenter erro, nec mihi hunc errorem quo delector dum vivo extorqueri volo.*"—*London Med. Times and Gazette.*

